# Diversity of soil fungi and entomopathogenic fungi in subtropical mountain forest in southwest China

**DOI:** 10.1111/1758-2229.13267

**Published:** 2024-06-29

**Authors:** Jiyang Zheng, Jinduo Shi, Dun Wang

**Affiliations:** ^1^ Key Laboratory of Crop Stress Biology for Arid Areas Northwest A&F University Yangling Shaanxi China; ^2^ Forest Bureau of Ankang City Ankang Shaanxi China

## Abstract

Till now, the diversity of entomopathogenic fungi in subtropical mountain forest was less studied. Here, the vertical distribution of forest soil fungi, entomopathogenic fungi, and their environmental influencing factors in a subtropical mountain in western China were investigated. Soil samples were collected from four elevations in a subtropical forest in Shaanxi. The results indicated a greater richness of soil fungi at middle elevations and soil fungi were more even at low elevation. Soil pH, available iron, available potassium, total potassium, and available zinc were the most important influencing factors affecting this vertical distribution of fungi. Interestingly, the *Isaria* genus was predominant while *Metarhizium* and *Beauveria* showed decreasing abundance. The presence of *Isaria* showed a significant positive correlation with both total phosphorus and available iron, while, available zinc was negatively correlated. *Metarhizium* was influenced by elevation, pH, available phosphorus, and available copper and *Beauveria* was influenced by soil organic carbon, total nitrogen, total potassium, available potassium, and available zinc. Overall, as environmental factors affecting soil fungi, elevation, and plant species diversity were less important than soil physical and chemical properties. The virulence of isolated entomopathogenic fungi were tested against larvae of *Tenebrio molitor*, with mortality ranging from 31.11% to 100%. The above findings provide valuable data to deepen our understanding of the diversity of entomopathogenic fungi in subtropical mountain forests.

## Graphical abstract

Entomopathogenic fungi diversity vertically differed in a sub‐tropical forest in Hua‐long Mountains in southwest China. *Isaria* genus was dominant while *Metarhizium* and *Beauveria* showed in decreasing abundance. The presence of species from three genus showed very different correlation with environmental factors, especially the soil pH, available iron, available potassium, total potassium and available zinc. The virulence of isolated entomopathogenic fungi against larvae of *Tenebrio molitor* had mortality ranging from 31.11 % to 100 %.

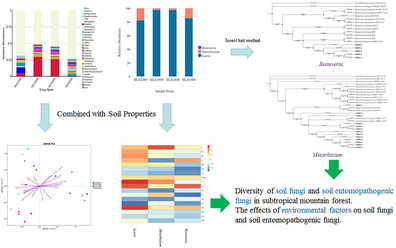

## INTRODUCTION

Changing environmental factors along altitudinal gradients are more obviously, and quickly observed, than those occurring along latitudinal gradients (Horuz et al., 2014). As this pertains to studies of biodiversity distribution patterns, this pattern is of paramount significance (Körnerb, 2007). Knowledge of altitudinal distribution patterns for soil microbial diversity is still in its infancy currently. Recently, relevant studies of biodiversity have focused primarily on the distribution patterns and the environmental factors that influence said distributions (Hallouti et al., 2020). Prior research has demonstrated that the study on soil microbial elevation‐based distribution patterns has made some progress with the development of sequence platforms. Globally, numerous elevation distribution patterns have appeared, though these could not be proven definitively as the factors that were driving the patterns varied by locality (Li & Ma, 2018). Certainly, understanding the factors influencing both patterns the distribution and diversity of fungi, both globally and regionally, along altitudinal gradients are key to understanding both the evolution and ecological role of fungi.

Soil microbes have integral ecological roles in nature, particularly as decomposer. Prior research has distinguished the ecological role of microbial groups, in fungal‐dominated soil, from that of bacteria (Brandan et al., 2017). Without soil fungi diversity and their associated activities, soil could not exist in on Earth (Chu et al., 2020). Soil fungi provide numerous nutrients for vegetation growth and simultaneously act as pathogens negatively affecting forest community health (Maron et al., 2011).

As aforementioned, global soil fungi distributions, within forest, varying along altitudinal and longitudinal gradients; this variability may be explained by the existence and influence of factors influencing said distributions. The presence of vegetation has a significant effect on elevational diversity patterns of soil fungi (Bryant et al., 2008), while vegetation type impacts soil fungal diversity and composition (Han et al., 2021), and fallen wood decay indirectly influences soil fungal diversity (Xu et al., 2023). Additionally, the physical and chemical properties of soil, such as soil pH, carbon, nitrogen, and soil elements (Hawkes et al., 2011; Wang et al., 2021), also have impacts on soil fungi distributions and diversity; while the concentration of soil nutrients influences fungal diversity most (Yang et al., 2022). Soil nutrients, such as carbon (C) and nitrogen (N), are the basic energy sources and constituent elements for soil fungi and can affect their distribution by influencing the metabolism process of soil fungi (Yang et al., 2014). In addition, soil micronutrients, such as copper (Cu), zinc (Zn), iron (Fe), and manganese (Mn), have a great influence on soil fungi, too. For example, Fe and Mn are used for fungal respiration, Cu and Zn are used for fungal immune activity, and Fe is used for nitrogen fixation (Dubinsky et al., 2010; Feng et al., 2019; Whalen et al., 2018). Therefore, soil nutrients and micronutrients can affect soil fungi greatly, such as microbial abundance and diversity (Dai et al., 2023). In conclusion, the soil physical and chemical properties have significant influences on the soil fungal community, furthermore, it can affect the composition, richness, and diversity of soil fungi. As these chemical and physical properties directly impact fungal communities they are of vital importance to better understanding the dynamic of soil fungi distributions and diversity. Elevation is particularly effective as a metric for quantifying environmental change over shorter scales. However, more broadly temperature, UV intensity, air pressure, and atmospheric oxygen content were all influenced by elevation, contributing to significant differences in the distribution of, and/or diversity within, soil fungal communities along altitudinal gradients (Descombes et al., 2017; Hodkinson, 2005; Sharma et al., 2021).

Entomopathogenic fungi are one of the most important groups in fungal community, as they play an important role in agricultural and forestry pest control. Entomopathogenic fungi in forests can kill host insects in the suitable conditions. The insect epidemics caused by entomopathogenic fungi are of great help in forest pest control (Dara et al., 2019). For example, in the outbreak areas of cockchafers (*Melolontha* spp.) in forest of Poland, the soil *Beauveria* could occur naturally (Niemczyk et al., 2019). In forests, insects infected by entomopathogenic fungi were frequently found, and these entomopathogenic fungi have higher biological control potentiality (Meng et al., 2022; Tang et al., 2021). Entomopathogenic fungi are widely distributed in different ecological environments even if pathogenic host insects are scarce (Araújo & Hughes, 2016; Kovač et al., 2021). Investigations into the factors influencing the distributions and diversity of entomopathogenic fungi are necessary due to increasing environmental change, such as the increased frequency of pest outbreak (Wang et al., 2022). Changing climatic conditions, and variable ecological environmental factors, have greatly effect on the structure of entomopathogenic fungi communities both directly and indirectly. Recently, studies have shown that an increasing number of novel entomopathogenic fungi were discovered, and subsequently isolated, with increasing comprehensive soil sampling (Wei et al., 2021). Entomopathogenic fungi, isolated from soil samples, ideally can be utilized in the pest management system. The ‘native’ entomopathogenic fungi are more adaptable to environmental conditions (Qayyum et al., 2021).

The HuaLong Mountain National Nature Reserve (HLMNNR) is located at the cross‐mountain region of Shaanxi Province, Hubei Province, and Chongqing, western China. The HLMNNR covers an area of 28,103 hectares with 91.7% forest coverage. The HLMNNR retains pristine forest ecosystems, an important resource pool of native animal and plant species in the northern range of Bashan mountains in west China. Furthermore, this natural complex has extremely high conservation value as it exemplifies the typical natural state of subtropical forest ecosystems therein. The HLMNNR has been called a ‘gene bank of biological species’, because of its dense forest, abundant biological resources, and vastly distributed rare species (Lv & Ling, 2017). This study in the HLMNNR was of import as it explored the integrated ecosystems within this expanse of subtropical forest.

In summary, the distribution of fungi and entomopathogenic fungi is related to soil, physical and chemical, properties, and geographic location (altitudinal and latitudinal gradients) (Fernandez‐Bravo et al., 2021; Quesada‐Moraga et al., 2007). The vertical distribution, and influencing factors for distribution, of forest soil fungi and entomopathogenic fungi in the HLMNNR were investigated utilizing high‐throughput sequencing technologies and isolation of entomopathogenic fungi. This work presented here sought to broaden the current understanding of the composition of soil fungi in the mountain forest ecosystems. The following hypotheses were proposed: (1) The composition and diversity of soil fungi and entomopathogenic fungi change significantly with the increase of elevation. (2) Apart from elevation and vegetation diversity, soil physical and chemical properties significantly influence the composition and diversity of soil fungi and entomopathogenic fungi. (3) Some virulent entomopathogenic fungi can be isolated from the soil.

## MATERIALS AND METHODS

### Location, survey, and samples collection

This study was carried out in July 2018 and July 2020 at the HLMNNR (31°54′39″–32°08′13″ N and 109°16′41″–109°30′29″ E, 894–2918 m above sea level). The mean anual temperature is 12.1°C. The mean annual precipitation is 1015 mm, with most of the rainfall distributed from May to September. The HLMNNR belongs to subtropical warm and humid mountainous climate zone. The HLMNNR basically maintains its originality and integrity with 91.7% forest coverage. The dominant vegetation type includes coniferous forest, broad‐leaved forest, shrub, and meadow. Forest soil types are classified as mountainous yellow soil and dark brown soil.

In this study, total 20 plots were set at four elevations (2200, 1800, 1400, and 1000 m). At each elevation, five independent replicate plots were established with a distance of above 1 km. Each plot was 10 m × 10 m quadrants, and five 1 m × 1 m sampling sites were chosen randomly in the same plot. One bag of soil sample was collected from one sample site. And then, five bags of soil samples collected in the same 10 m × 10 m quadrants were fully mixed into one packet to represent as one plot sample. Finally, 20 packets of soil sample were collected at four elevations. Approximately 500 g soil sample was collected of one packet. The first step of soil sample collection was to remove the organic layer above the soil in order to collect the soil samples from 10 to 20 cm depth for each sub‐plot. Then a 2‐mm screen was used to remove residual debris and roots, and soil sample were put into a new soil sample packet. Finally, all soil samples were transported to the laboratory at sustained −20°C. The collected soil samples and environmental factors at different elevations were used for a correlation analysis for the key factors and fungal diversity. The soil samples from each plot were subject to the high‐throughput sequencing, determination of soil physical–chemical properties, and the entomopathogenic fungi isolation as described in the following Sections Environmental factors collection, Isolation of soil entomopathogenic fungi.

### Environmental factors collection

Elevation (E), herb species diversity (HS), and woody species diversity (WS) were recorded at each of the 20 plots (Table S1). At the same time of soil sample collection, environmental factors was immediately investigated and recorded. As mentioned above, each soil sampling plot was 10 m × 10 m quadrants, and five 1 m × 1 m sampling sites were chosen randomly in the same plot. One bag of soil sample was collected from one sample site. And then, five bags of soil samples collected in the same 10 m × 10 m quadrants were fully mixed into one packet to represent as one plot sample. The different herb species number and different woody (arboreal and shrubby) species number in each 10 m × 10 m quadrants were recorded, representing herb species diversity and WS diversity respectively in each soil sampling plot. The investigation method described by Liang et al. (2016) was used with minor modification. For the arboreal layer, the number of different WS with DBH (diameter at the breast height) ≥ 1 cm was recorded. For the shrubby and herbaceous layers, the total number of different species was recorded. Identification of different vegetation species was carried out morphologically based on the National Plant Specimen Resource Center (http://www.cvh.ac.cn). Each soil sampling plot was corresponded to a series of environmental factor data including E, HS, and WS.

### DNA extraction, amplification, and sequencing of high‐throughput sequencing

Soil total genomic DNA were extracted from 10 g soil sample by using the method described by Bürgmann et al. (2001) with some modifications. DNA preliminary extracts were checked on 1.5% agarose gels at 80 V in TAE buffer (EDTA: 2 M, pH 8.0 and Acetic acid: 0.02 M; Tris base: 0.04 M) and the correct strip area was purified with a DNA Extraction Kit (Beijing Solarbio Science&Technology Co., Ltd). The DNA samples were sent to Novogene (Beijing, China) for analysis using high‐throughput sequencing. Fungal ITS2 conservative region was PCR‐amplified using primers ITS3‐2024F (5′‐GCATCGATGAAGAACGCAGC‐3′) and ITS4‐2409R (5′‐TCCTCCGCTTATTGATATGC‐3′) (Alberto et al., 2012). Purified amplicons were sequenced on the IonS5™XL platform by Novogene (Beijing, China).

### Soil analysis

Each soil sample was determined for soil physical properties such as soil moisture content (SMC). Several normal soil nutrient elements such as soil organic carbon (SOC), total nitrogen (TN), soil pH, total phosphorus (TP), available phosphorus (AP), total potassium (TK), and available potassium (AK) were determined respectively. Some metal elements such as available copper (ACu), available zinc (AZn), available iron (AFe), available manganese (AMn), exchangeable calcium (ECa), and exchangeable magnesium (EMg) were focused in this study, too.

About 50 g of each sample was collected after pretreatment. The soil moisture determination used about 3 g soil sample by being oven‐dried at 105 ± 2°C for about 12 h. In this study, all soil properties were determined using standard procedures (Bao, 2000). Soil pH was determined by PB‐10 pH metre (Sartorius Company) after being mixed to a soil water slurry and the soil‐to‐water ratio was 1: 2.5. Determination of SOC was used TOC‐VSeries SSM‐5000A (Solid Sample Mearsurement). TN was determined by Kjeldahl Nitrogen Analyser (Haieng Future Technology Group Co., Ltd.) after digesting by H_2_SO_4_ according to Kjeldahl method. TP was determined by anti‐colour of molybdenum and antimony on UV‐1900i (Japan Shimadzu Company) after melting soil samples with NaOH. TK was determined by flame photometric detection on FP6410 Flame Photometer (Shanghai Instrument and Electrical Analysis Instrument Co., Ltd.) after melting soil samples with NaOH. AP was determined by molybdenum antimony colorimetric method on UV‐1900i (Japan Shimadzu Company) after 0.5 M NaHCO_3_ extraction. AK was determined by flame photometry on FP6410 Flame Photometer (Shanghai Instrument and Electrical Analysis Instrument Co., Ltd.) after 1 M NH_4_OAc extraction. Four soil‐available metal elements: ACu, AZn, AFe, and AMn were determined by atomic absorption spectrophotometry on Hitachi Z2000 Atomic Absorption Spectrophotometer (Japan Hitachi Limited) after hydrochloric acid or DTPA‐TEA extraction at same time. ECa and EMg was determined on Hitachi Z2000 Atomic Absorption Spectrophotometer (Japan Hitachi Limited) by atomic absorption method.

All data of determined soil properties in this study were mentioned in Supporting information Table S1.

### Isolation of soil entomopathogenic fungi

Entomopathogenic fungi were isolated from soil samples by the insect bait method (Zimmermann, 1986). We used the *Tenebrio molitor* (Coleoptera: Tenebrionidae) raised at Lab of Insect Relative Resource (College of Plant Protection, Northwest A & F University, Shaanxi Province, Northwestern China) to isolate entomopathogenic fungi. Ten healthy larvae of seventh or eighth instar (1.2–1.5 cm) filled with 100 g soil were put into a 500 mL plastic box, then the soil in plastic box was moistened with sterilized water and maintained at 25 ± 2°C. Importantly, all plastic boxes were rotated three times every day so as to impel the insect bait to move through the soil samples, it helped to increase the possibility they would expose to potential pathogenic fungi in the soil samples. The dead larvae were checked and removed in every 2 days. Larvae were checked with sterilized tweezers, and dead larvae with hard body or covered by fungi were transferred to a 24‐well plastic boxes with a sterilized filter paper moistened by sterilized ddH_2_O, in order to determine whether it was a fungal infection and helped the fungi to grow out the dead larvae. The 24‐well plastic boxes contained dead larvae were maintained at 25 ± 2°C. After the fungi grew out of the dead larvae, the dead larvae were surface‐sterilized in 75% alcohol respectively, and then they were plated on semi‐selective agar medium. The semi‐selective agar medium composed of 10 g/L peptone, 10 g/L yeast extract, 40 g/L d‐glucose anhydrous, 10 g/L agar, 0.6 g/L cetyltrimethyl ammonium bromide (CTAB), 0.6 g/L streptomycin, 0.6 g/L kanamycin was utilized to isolate entomopathogenic fungi (Posadas et al., 2012). All plates were incubated for 14 days at 25 ± 2°C in darkness. Then, the desired colony was transferred to 1/4 SDAY medium by using sterilized toothpicks. The 1/4 SDAY medium was composed of 2.5 g/L peptone, 2.5 g/L yeast extract, 10 g/L d‐glucose anhydrous, 10 g/L agar, 0.6 g/L streptomycin, 0.6 g/L kanamycin.

During the soil samples collection, all naturally infected insects (from Cercopidae and Plataspidae) found in HLMNNR were also collected for later isolation of entomopathogenic fungi. The infected larvae were isolated by the semi‐selective agar medium (Posadas et al., 2012) after surface sterilization with 75% ethanol, and cultured in an artificial incubator at 25°C for 14 days. Then the edges of the colonies were selected and purified in the 1/4 SDAY medium.

### Fungal genomic DNA extraction, PCR amplification and sequencing

Genomic DNA of entomopathogenic fungi was extracted by using the method described by Aljanabi and Martinez (1997) with minor modifications. The isolated entomopathogenic fungus was inoculated to SDAY medium respectively for 14 days at 25 ± 2°C to culture mycelia. Then about 100 mg mycelia was collected into a 1.5 mL tube. 650 μL of TE buffer (10mMTris‐HCl; 1 mM EDTA, pH = 8.0), 100 μL of 10% SDS solution, and 20 μL of protenase K (10 mg/mL) were added in it, incubated at 55°C for at least 5 h or overnight. After that, 700 μL of 6 M NaCl solution was added, and it was vortexed for 30 s. 750 μL of supernatant was transferred to a new 1.5 mL tube after 30 min centrifugation at 12000 g. Then 750 μL of of isopropanol was added, mixed, and incubated at −20°C for at least 1 h or overnight. After that, it was centrifuged at 12000 g, 4°C for 20 min. The supernatant was poured out, and 70% ethanol was added to wash the precipitate in the tube. Finally, the precipitate was dried and resuspended in 50 μL of TE buffer. The extracted DNA was stored at −20°C until use. The DNA was applied as a template in order to amplify the target genes by PCR (polymerase chain reaction) amplification. The PCR amplification was performed in 50 μL volumes, which contained 2 μL DNA template, 1 μL each primer, 25 μL PCR mixture (GenStar, Beijing, China), and 21 μL twice‐sterilized water. The internal transcribed spacers (ITS) region was amplified using the primer ITS1F (5′‐TCCGTAGGTGAACCTGCGG‐3′) and ITS4R (5′‐TCCTCCGCTTATTGATATGC‐3′) (White et al., 1990). The large subunit ribosomal RNA (nr*LSU*) region were amplified using the primer LR5 (5′‐ATCCTGAGGGAAACTTC‐3′) and LR0R (5′‐GTACCCGCTGAACTTAAGC‐3′) (Vilgalys & Sun, 1994). And the elongation factor 1 alpha (*EF1α*) region was amplified using the primer EF1α‐EF (5′‐GCTCCYGGHCAYCGTGAYTTYAT‐3′) and EF1α‐ER (5′‐ATGACACCRACRGCRACRGTYTG‐3′) (Bischoff et al., 2005). Then, the PCR was performed separately, and the PCR products were sequenced by sequencing company (Sangon Biotech, Shanghai, China).

### Phylogenetic analysis

All obtained sequences were aligned with available data by BLAST from the Gen‐Bank database (NCBI, https://www.ncbi.nlm.nih.gov/) first (Zhou et al., 2020). We used MEGA X to align each sequence for same the genus. Then, the cladogram was formed by maximum likelihood analyses in PhyloSuite v1.2.1 (Lanfear et al., 2017; Nguyen et al., 2015; Zhang et al., 2020). For all partitions, a Bayesian information criterion model was used. Nodal support was assessed with nonparametric bootstrap using 1000 pseudo‐replicates.

### Virulence of isolated entomopathogenic fungi

In order to prove the pathogenicity of isolated fungi to insects, we conducted a bioassay. To obtain conidia, each fungus was incubated on 1/4 SDAY for 14 days at 25 ± 2°C with total darkness. Conidia were harvested with sterilized wood chips, added to sterilized water containing 0.05% Tween‐80, and vortexed for 5 min. Through an inverted research microscope (ECLIPSE TE2000‐S, Nikon, Tokyo, Japan), the spore suspension concentration was adapted to 10^6^ conidia/ml and 50 mL by haemocytometer under 10× or 40× magnification. The sterilized water containing 0.05% Tween‐80 was used for the control group.

In this study, the tested insects were *Tenebrio molitor*. The healthy larva of eighth instar was selected for each fungus in 3 groups of 15. The tested insects were immersed completely in suspension of each fungus with constant agitation. Then the treated insects were transferred to 24‐well plastic boxes ventilated with several holes. All the plastic boxes were stored at standard environmental chamber conditions (25 ± 2°C, 60% ± 5% RH, and 12:12 L:D photoperiod). Wheat bran was used to feed insects during the bioassay. Insect mortalities were recorded every day for 7 consecutive days.

### Statistic analysis

After soil total genomic DNA extracted and ITS2 region PCR‐amplified, purified amplicons were sequenced on the IonS5™XL platform by single reads. We used Cutadapt (V1.9.1) (Shuo et al., 2016) to cut the low‐quality part of the reads, and then according to Barcode, the data was separated from the obtained reads. Trimmomatic (version 0.33) (Bolger et al., 2014) was used to filter for sequencing errors. After preliminary quality control of Barcode and primer sequences, the original data (Raw data) were obtained. Then, the Raw data was spliced and filtered to obtain Clean data by removing chimeric sequences, detecting chimera sequences on Species Annotation Database (https://github.com/torognes/vsearch/) (Qin et al., 2012) and removing the chimera sequence therein (Nguyen et al., 2016).

USEARCH (version 10.0) (Edgar, 2013) was used to cluster sequences at a similarity of 97% (default) and verification of sequence origin. All Clean data we got were clustered, and with the identity of 97%, sequences were clustered into operational taxonomic units (OTUs). About singletons, OTUs were filtered by a threshold of 0.005% of the number of all sequenced sequences. Then, the Species annotation analysis was performed by the Blast of Qiime (Version 1.9.1) (Rognes et al., 2016) aligned with Unit Database (v7.2) (Haas et al., 2011). At each classification level, the community composition was analysed. Finally, homogenization was carried out with the minimum amount of data in the sample as the standard.

After that, the Alpha diversity indices including Chao1 index, ACE index, Shannon index, and Simpson index were estimated by Qiime (Version 1.9.1) (DeSantis et al., 2006) and displayed with R package (Version 2.15.3). The Dilution curve was made by R package (Version 2.15.3). For Beta diversity, we used Qiime (Version 1.9.1) to calculate Unifrac distance (Li et al., 2013), the principal co‐ordinates analysis (PCoA) was performed to explain the Beta diversity by Weighted Gene Correlation Network Analysis, stats, and ggplot2 of R package.

In order to explore the relationship between soil fungal community composition and environmental factors, distance‐based Redundancy Analysis (dbRDA) (Sheik et al., 2012) and Spearman correlation analysis (Clarke, 2010) were performed. For the dbRDA analysis, the envfit function was used to calculate the r2 and *p*‐values of each environmental factor, and then the environmental factors with significant effects were selected and analysed using the rda function in the vegan package. Because there were many unpredictable variables in soil fungi and soil properties at different soil samples and some ‘outliers’ might be crucial, the Spearman correlation coefficient was chosen and calculated by the corr.test function of the psych package in R package (Version 2.15.3) and its significance was tested at the same time. Then the pheatmap function was used for visualization in the pheatmap package.

## RESULTS

### The variation in soil fungi at different elevations

#### Soil fungal composition

**FIGURE 1:**
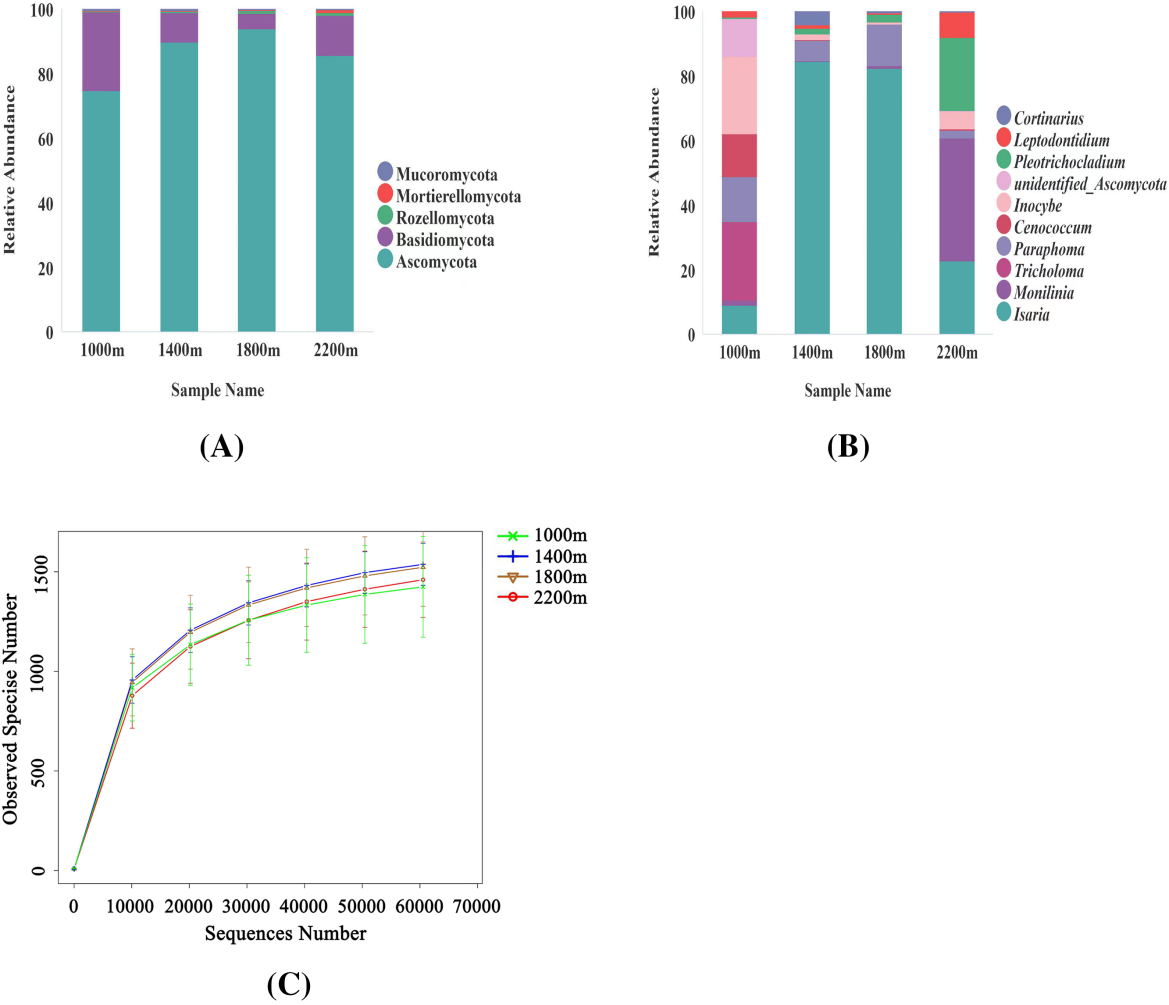
The fungal relative abundance histogram and rarefaction curve. (A) Relative abundance histogram of top 5 fungal phylum; (B) Relative abundance histogram of top 10 fungal genera. (C) The rarefaction curve. The relative abundance of top 5 phylum fungi referred to the 5 phylum with the highest relative abundance. (The relative abundance was the proportion of one species relative to all species in the corresponding sample, which was the ratio of the number of sequences corresponding to this species to the number of sequences corresponding to all species.) The relative abundance of top 10 genera fungi referred to the 10 genera with the highest relative abundance. 1000 m, elevation of 1000 m in HLMNNR; 1400 m, elevation of 1400 m in HLMNNR; 1800 m, elevation of 1800 m in HLMNNR; 2200 m, elevation of 2200 m in HLMNNR.

A total of 1,614,585 high‐quality sequences after quality control were obtained by high‐throughput sequencing of the ITS2 region. A total of 5558 OTUs (>97% sequence similarity level) were clustered, and 277 OTUs were unassigned. And the depth of the sequence was shown by the rarefaction curve in Figure 1C. The 5558 OTUs were assigned to 126 orders, 287 families, 683 genera, and 725 species. The top 5 relative abundance on phylum were shown (Figure 1A) according to the species annotation results. Ascomycota (average 62.22% of total OTUs) was clearly predominant, followed by Basidiomycota (average 9.09% of total OTUs). And there was little difference among four elevations. The top 10 relative abundance on genus were shown (Figure 1B) and the top 30 fungal relative abundance on genus of different soil samples were shown on Figure S1 according to the species annotation result. Interestingly, *Isaria* (average 27.59% of total OTUs) was predominant, especially in 1800 and 1400 m. However, *Monilinia* (8.65%), followed by *Pleotrichocladium* (5.18%) and *Isaria* (5.14%) were predominant at 2200 m. While the elevation of 1000 m showed the largest soil fungal evenness and *Tricholoma* (3.37%) had the highest relative abundance.

#### Soil fungal diversity

The fungal community evenness and richness were shown by Alpha diversity index (Table 1) based on clustered OTUs. Overall, these results indicated that the richness of soil fungi had no especially obvious pattern along elevation. However the richness of soil fungi followed a gradually unimodal pattern with a peak at 1400 m. The evenness of soil fungi had no obvious pattern along elevation, too. While the evenness of soil fungi was largest at 1000 m.

**TABLE 1:** The Alpha diversity indices (Mean ± SE).

Group	Observed species	Shannon	Simpson	chao1	ACE
1000 m	1424.400 ± 126.398	7.701 ± 0.297	0.983 ± 0.004	1531.425 ± 139.860	1540.699 ± 137.572
1400 m	1537.400 ± 52.565	7.028 ± 0.663	0.922 ± 0.044	1651.603 ± 54.955	1660.667 ± 51.917
1800 m	1523.000 ± 98.173	6.716 ± 0.735	0.897 ± 0.048	1643.231 ± 95.673	1648.147 ± 96.373
2200 m	1460.200 ± 94.612	6.845 ± 0.524	0.941 ± 0.032	1593.578 ± 86.434	1611.766 ± 82.702

*Note*: The Alpha diversity of the fungal community with different elevation in HLMNNR at 97% similarity. 1000 m, elevation of 1000 m in HLMNNR; 1400 m, elevation of 1400 m in HLMNNR; 1800 m, elevation of 1800 m in HLMNNR; 2200 m, elevation of 2200 m in HLMNNR.

**FIGURE 2:**
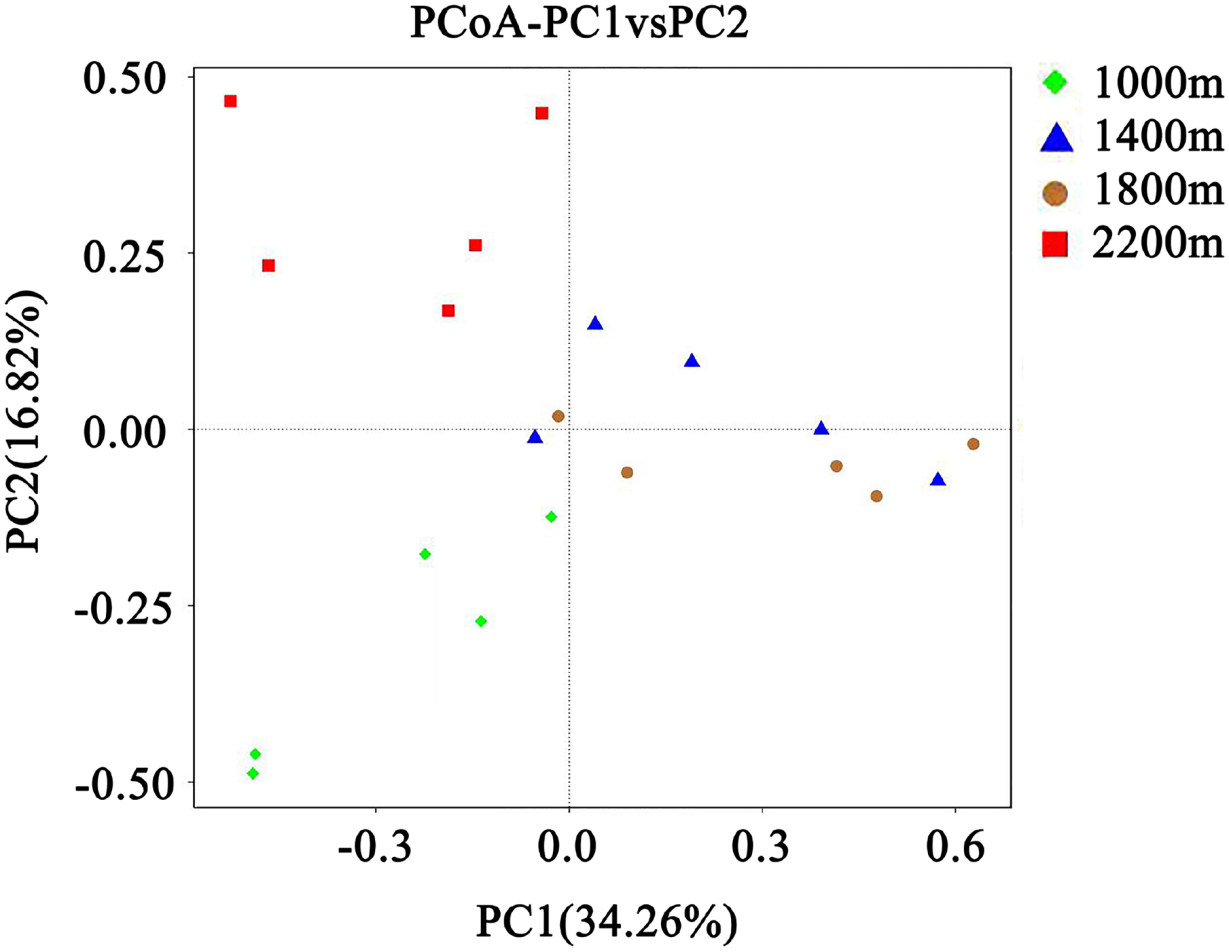
The PCoA analysis. The PCoA analysis based on the UniFrac distance of the soil fungal community with different elevation in HLMNNR. 1000 m, elevation of 1000 m in HLMNNR; 1400 m, elevation of 1400 m in HLMNNR; 1800 m, elevation of 1800 m in HLMNNR; 2200 m, elevation of 2200 m in HLMNNR.

The PCoA analysis was performed to reflect fungal Beta diversity (Figure 2), and significant differences among elevation were shown. The geographic distances of different elevations were found dissimilar in this study. In the PCoA analysis, the distance between 1400 m and 1800 m was relatively small, indicating that the fungal community composition in 1400 and 1800 m were found more similar. Taken together, these results demonstrated that there were three distinct patterns of fungal distribution along the elevation in the HLMNNR.

#### Responses of soil fungi to environmental factors

**FIGURE 3:**
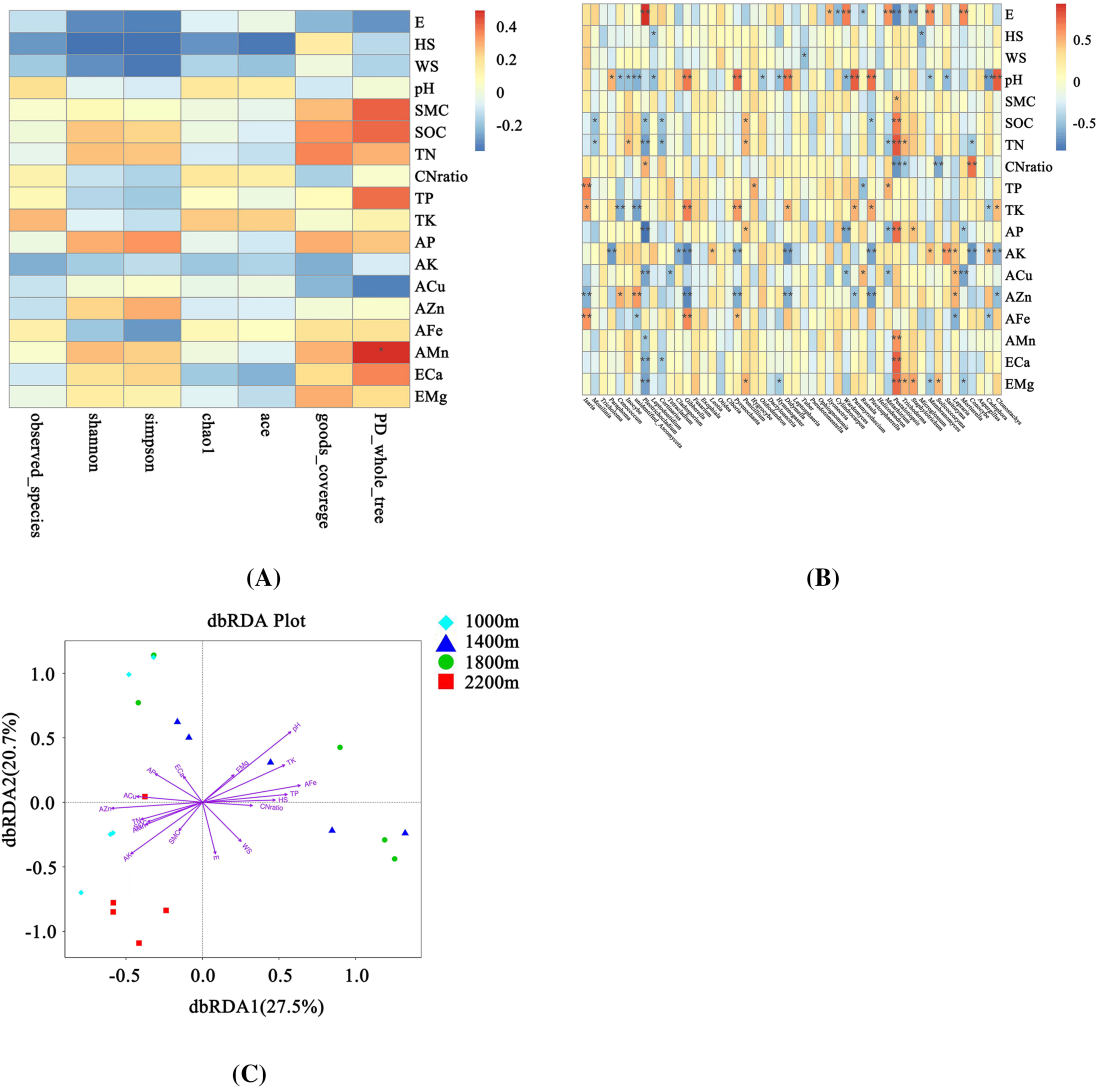
The dbRDA analysis and spearman analysis. (A) The spearman analysis of environmental factor's influence on the soil fungal Alpha diversity index. (B) The spearman analysis of environmental factor's influence on the top 50 genus fungi. (C) The dbRDA analysis. The dbRDA analysis was performed to reflect and compare the every environmental factors' influence on the soil fungal community composition. The spearman analysis was performed to reflect and compare the every environmental factor's influence on the soil fungal Alpha diversity index (A), and influence on the top 50 genus fungi (B). The independent observable variables were elevation (E), herb species diversity (HS), woody species diversity (WS), and soil properties (including soil pH, SMC, SOC, TN, TP, TK, AP, AK, ACu, AZn, AFe, AMn, ECa, EMg). ** represented that *p* <0.01. * represented that *p*<0.05. HLS1000, elevation of 1000 m in HLMNNR; HLS1400, elevation of 1400 m in HLMNNR; HLS1800, elevation of 1800 m in HLMNNR; HLS2200, elevation of 2200 m in HLMNNR.

The dbRDA analysis was performed to reflect and compare the every environmental factor's influence on the soil fungal community composition (Figure 3C). Furthermore, the RDA1 and RDA2 axis of dbRDA explained 27.5% and 20.7%, respectively. PH (*r*2 = 0.63), AFe (*r*2 = 0.42), AK (*r*2 = 0.38), TK (*r*2 = 0.37), AZn (*r*2 = 0.35) were found more important impacting the soil fungal community composition (*p*< 0.05). The influence of E (*r*2 = 0.17) on the soil fungal community composition was less influential relatively. Similarly, HS (*r*2 = 0.22) and WS (*r*2 = 0.15) were also less influential than soil physical and chemical properties.

Spearman correlation analysis was performed to reflect and compare the every environmental factor's influence on the soil fungal Alpha diversity index (Figure 3A). Elevation and vegetation species diversity had negative influence on soil fungal evenness and richness. Not the same, the influences of soil physical and chemical properties on soil fungal evenness and richness were various. Furthermore, the interactions between environmental factors and top 50 genus were investigated by Spearman correlation analysis (Figure 3B), too. E and *Pleotrichocladium* was found significant correlation (*r* = 0.93, *p*<0.01) and E had a positive influence on *Pleotrichocladium*. On the contrary, there was a negative influence (*r* = −0.82) on *Pleotrichocladium* by AP. In addition to *Pleotrichocladium*, *Pestalotiopsis* was also greatly effected by environmental factors. Overall, HS, WS, and SMC had less impact on top 50 genus of fungi, and the dominant factors were pH, AK, and AZn. Some environmental factors had opposite influence on the fungi, such as AZn and AFe. While some environmental factors had same influence on the fungi, such as AZn and AK.

### The variation in soil entomopathogenic fungi at different elevations

#### Soil entomopathogenic fungal composition and diversity

**FIGURE 4:**
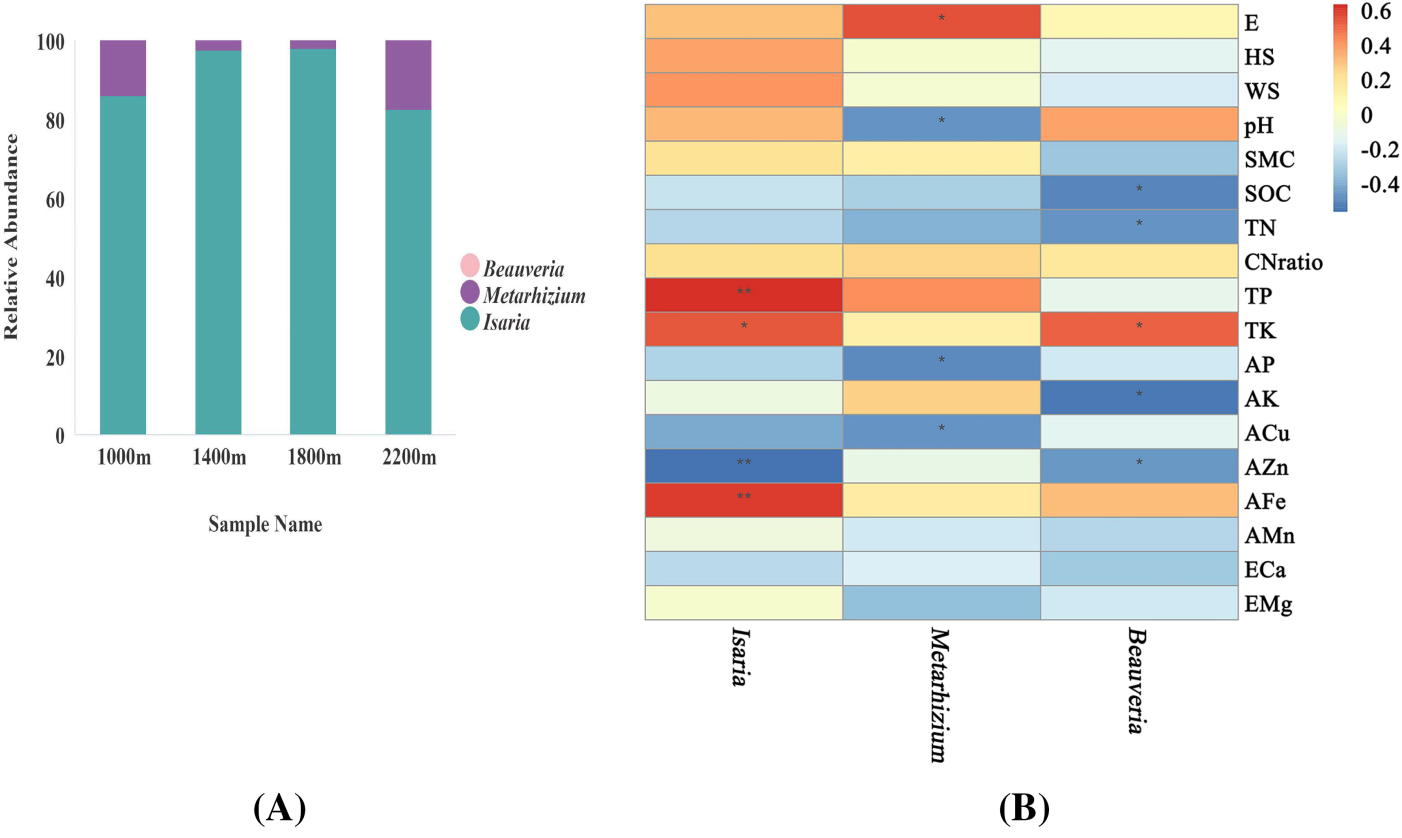
The fungal relative abundance histogram and spearman analysis. (A) Relative abundance of entomopathogenic fungi at genus level. (B) The spearman analysis of environmental factors' influence on the soil entomopathogenic fungi. The relative abundance of entomopathogenic fungi was obtained through high‐throughput sequencing. The spearman analysis was performed to reflect and compare the every environmental factors' influence on the soil entomopathogenic fungi. The independent observable variables were elevation (E), herb species diversity (HS), woody species diversity (WS), and soil properties (including soil pH, SMC, SOC, TN, TP, TK, AP, AK, ACu, AZn, AFe, AMn, ECa, EMg). ** represented that *p*<0.01. * represented that *p*<0.05. HLS1000, elevation of 1000 m in HLMNNR; HLS1400, elevation of 1400 m in HLMNNR; HLS1800, elevation of 1800 m in HLMNNR; HLS2200, elevation of 2200 m in HLMNNR.

The relative abundance of entomopathogenic fungi on genus were shown (Figure 4A), and the entomopathogenic fungal relative abundance on genus of different soil samples were shown on Figure S2 according to the species annotation results. *Isaria* and *Metarhizium* were predominant at genus. And *Isaria* was clearly predominant, followed by *Metarhizium*. The relative abundance of *Beauveria* was lowest among *Isaria*, *Metarhizium*, and *Beauveria*.

The entomopathogenic fungal community evenness and richness were shown (Table 2). The richness of soil fungi had decreasing pattern along elevation. The trend of entomopathogenic fungi was clearly different from fungi. The richness and evenness of entomopathogenic fungi in 1000 m were relatively higher compared to elevation 1400, 1800, and 2200 m. And soil entomopathogenic fungal evenness trended to be lowest in 1400 m. The variation of Alpha diversity of soil entomopathogenic fungi at four elevations was not the completely same as soil fungi (Table 1).

**TABLE 2:** The Alpha diversity of entomopathogenic fungal community richness and diversity (Mean ± SE).

Group	Observed species	Shannon	Simpson	chao1	ACE
1000 m	8.600 ± 0.872	1.878 ± 0.190	0.583 ± 0.052	12.800 ± 3.441	15.890 ± 4.909
1400 m	5.000 ± 0.707	1.197 ± 0.115	0.407 ± 0.028	5.600 ± 1.077	7.018 ± 2.106
1800 m	6.200 ± 1.562	1.559 ± 0.349	0.514 ± 0.090	6.200 ± 1.562	6.800 ± 1.522
2200 m	5.200 ± 0.583	1.568 ± 0.083	0.555 ± 0.029	5.200 ± 0.583	5.634 ± 0.694

*Note*: The Alpha diversity of the fungal community with different elevation in HLMNNR at 97% similarity. 1000 m, elevation of 1000 m in HLMNNR; 1400 m, elevation of 1400 m in HLMNNR; 1800 m, elevation of 1800 m in HLMNNR; 2200 m, elevation of 2200 m in HLMNNR.

#### Responses of soil entomopathogenic fungi to environmental factors

Spearman correlation analysis was performed to reflect and compare the every environmental factor's influence on the soil entomopathogenic fungi (Figure 4B). Most importantly, *Isaria* had the highest relative abundance on genus, TP (*r* = 0.64) and AFe (*r* = 0.60) were found positively correlated with it (*p*<0.01), while AZn (*r* = −0.56) was found negatively correlated with it (*p*<0.01). E (*r* = 0.55), pH (*r* = −0.47), AP (*r* = −0.50), ACu (*r* = −0.47) were found correlation with *Meatrhizium* (*p*< 0.05) and SOC (*r* = −0.51), TN (*r* = −0.47), TK (*r* = 0.52), AK (*r* = −0.55), AZn (*r* = −0.47) were found correlation with *Beauveria* (*p*< 0.05). Non‐soil factors such as elevation, and vegetation diversity had less impact on entomopathogenic fungi than physical and chemical properties.

#### Entomopathogenic fungi isolation

**FIGURE 5:**
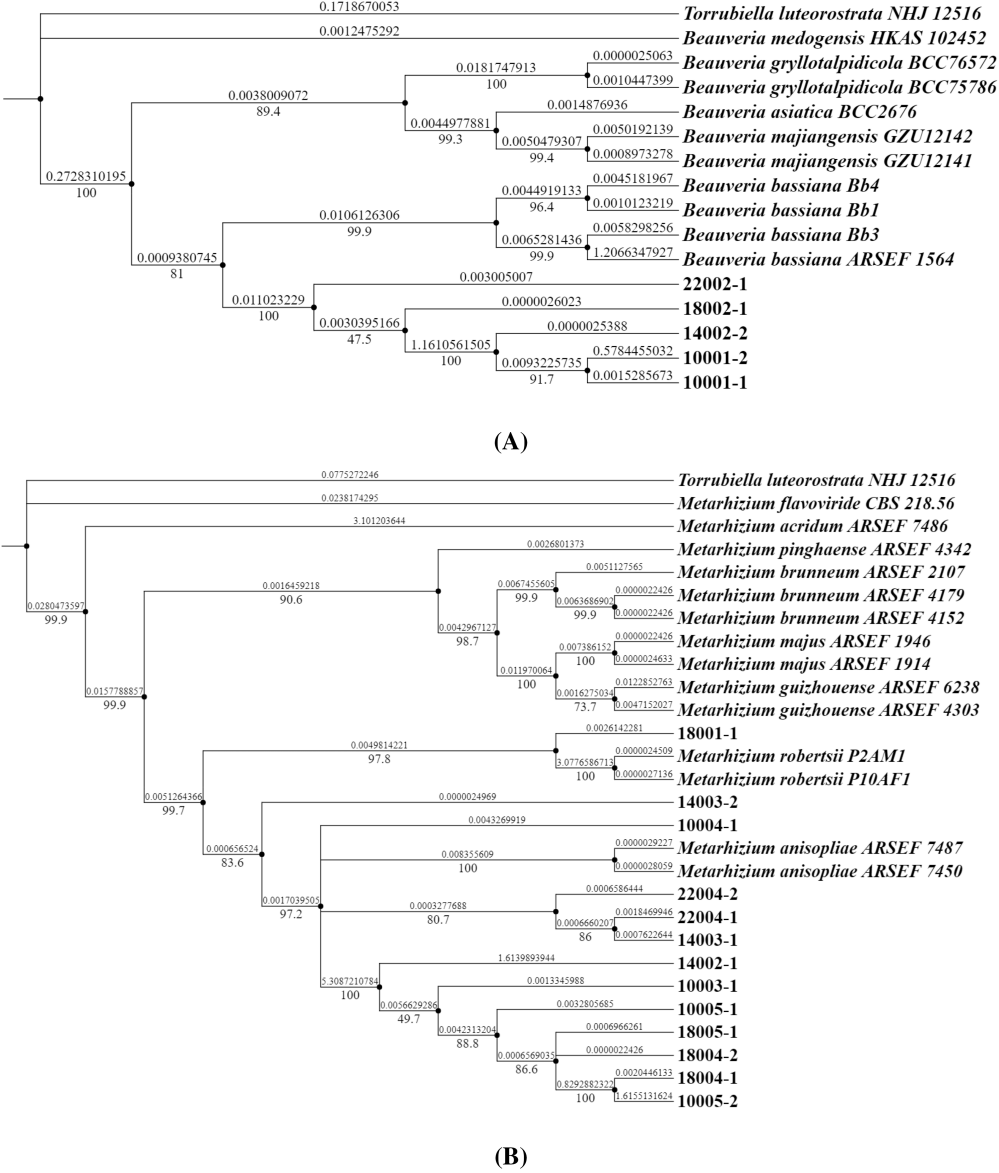
Maximum likelihood (ML) phylogenetic tree of fungal isolates. (A) *Beauveria* strains. (B) *Metarhizium* strains. The tree was based on *ITS*, *EF‐1α*, and *nrLSU* sequences by using Phylosuite v1.2.1. and rooted by using *Torrubiella luteorostrata NHJ12516* as outgroup.

A total of 13 *Metarhizium* isolates and 5 *Beauveria* isolates were isolated from 20 soil samples in this study. In addition, 2 *Isaria* isolates was isolated from infected insects in HLMNNR and published previously (Tang et al., 2021). The number of *Metarhizium anisopliae* isolates was 12 and were the most, followed by *Beauveria bassiana* (5 isolates), *Isaria farinosa* (2 isolates), *Metarhizium robertsii* (1 isolate) (Table 3). The phylogenetic tree of *Beauveria* (Figure 5A) and *Metarhizium* (Figure 5B) was established with *Torrubiella luteorostrata NHJ12516* as the outgroup (Figure 5). After morphological identification, we identified the species of isolated entomopathogenic fungi ultimately (Table 3). The identification of *Isaria farinosa* (2 isolates) were published and shown previously (Tang et al., 2021).

**TABLE 3:** Identification result of isolated entomopathogenic fungi of different entomopathogenic fungi isolated from 20 soil samples in HLMNNR.

Name	Identification result	Source
10001‐1	*Beauveria bassiana*	Soil
10001‐2	*Beauveria bassiana*	Soil
10003‐1	*Metarhizium anisopliae*	Soil
10004‐1	*Metarhizium anisopliae*	Soil
10005‐1	*Metarhizium anisopliae*	Soil
10005‐2	*Metarhizium anisopliae*	Soil
14002‐1	*Metarhizium anisopliae*	Soil
14002‐2	*Beauveria bassiana*	Soil
14003‐1	*Metarhizium anisopliae*	Soil
14003‐2	*Metarhizium anisopliae*	Soil
18001‐1	*Metarhizium robertsii*	Soil
18002‐1	*Beauveria bassiana*	Soil
18004‐1	*Metarhizium anisopliae*	Soil
18004‐2	*Metarhizium anisopliae*	Soil
18005‐1	*Metarhizium anisopliae*	Soil
22002‐1	*Beauveria bassiana*	Soil
22004‐1	*Metarhizium anisopliae*	Soil
22004‐2	*Metarhizium anisopliae*	Soil
HLS	*Isaria farinosa*	Infected insect of Cercopidae
HLS2	*Isaria farinosa*	Infected insect of Plataspidae

*Note*: 1000, elevation of 1000 m in HLMNNR; 1400, elevation of 1400 m in HLMNNR; 1800, elevation of 1800 m in HLMNNR; 2200, elevation of 2200 m in HLMNNR.

Total three genera of *Beauveria*, *Metarhizium*, and *Isaria* were obtained from isolation. And it was also found in high‐throughput sequencing result (Figure 4A). However, *Isaria* was unable to be isolated from soil samples in lab condition due to its biological character but was successfully isolated from infected insects from the same forest (Tang et al., 2021).

#### Virulence of isolated entomopathogenic fungi

All isolated entomopathogenic fungi used in the bioassay were pathogenic to *Tenebrio molitor* larvae, but the virulence was different (Table 4). The lowest virulence of isolated entomopathogenic fungi was 31.11% ± 2.22%, and the highest virulence of isolated entomopathogenic fungi was 100%. Although there were some differences in virulence, the isolated entomopathogenic fungi was proved pathogenic to insects clearly.

**TABLE 4:** The corrected mortalities (%) of entomopathogenic fungi against *Tenebrio molitor* for 7 days.

Isolate number	Identification result	Corrected mortalities (%)
10001‐1	*Beauveria bassiana*	64.44 ± 5.88
10001‐2	*Beauveria bassiana*	75.56 ± 2.22
10003‐1	*Metarhizium anisopliae*	62.22 ± 8.01
10004‐1	*Metarhizium anisopliae*	91.11 ± 8.89
10005‐1	*Metarhizium anisopliae*	66.67 ± 10.19
10005‐2	*Metarhizium anisopliae*	66.67 ± 11.55
14002‐1	*Metarhizium anisopliae*	55.56 ± 5.88
14002‐2	*Beauveria bassiana*	88.89 ± 5.88
14003‐1	*Metarhizium anisopliae*	82.22 ± 2.22
14003‐2	*Metarhizium anisopliae*	100
18001‐1	*Metarhizium robertsii*	75.56 ± 2.22
18002‐1	*Beauveria bassiana*	73.34 ± 6.67
18004‐1	*Metarhizium anisopliae*	64.44 ± 9.69
18004‐2	*Metarhizium anisopliae*	60 ± 13.33
18005‐1	*Metarhizium anisopliae*	100
22002‐1	*Beauveria bassiana*	35.56 ± 2.22
22004‐1	*Metarhizium anisopliae*	100
22004‐2	*Metarhizium anisopliae*	84.44 ± 2.22
HLS	*Isaria farinosa*	31.11 ± 2.22
HLS2	*Isaria farinosa*	33.33 ± 6.67

*Note*: 1000, elevation of 1000 m in HLMNNR; 1400, elevation of 1400 m in HLMNNR; 1800, elevation of 1800 m in HLMNNR; 2200, elevation of 2200 m in HLMNNR.

## DISCUSSION

### The variation in soil fungi

In this study, we found that the community composition and diversity of soil fungi were different in four elevations. Similarly, Ascomycota and Basidiomycota were the dominant phyla in the HLMNNR, not change with elevation, and it was similar to findings on the related studies of Ascomycota and Basidiomycota (Baldrain et al., 2021). The dominance of Ascomycota and Basidiomycota was established according to the abundance of wind‐dispersed spores, their biological characters, and functions (Viscarra Rossel et al., 2022). For example, the dominance of Basidiomycota in soil was related to its ability of degrading complex lignocellulose components as there was a thick litter layer normally in the forest soil (Lundell et al., 2010). The most important and interesting result of this study was that *Isaria* was the dominant genus in the HLMNNR. Even the isolation was failed to isolate *Isaria* from the soil samples, several *Isaria* species were successfully isolated from some infected insects samples from HLMNNR (Tang et al., 2021). This isolation result was consistent with previous studies that regular isolation hardly be successful to isolate *Isaria* from forest soil samples (Meyling et al. 2011; Steinauer et al. 2016). However, some previous studies found less *Isaria* species found dominant including tropical forest (Vivekanandhan et al., 2020), sub‐tropical forest (Sun & Liu, 2008), and temperate forest (Deaver et al., 2019; Sun & Liu, 2008). Therefore, the dominance of *Isaria* in HLMNNR might be a result of specific environmental condition, and further study was needed to discover its mechanism.

Our results indicated that the richness of fungi increased first and then decreased along elevation, while the diversity of fungi decreased first and then increased. On the contrary, Liu et al. (2018) found that the fungi species diversity and richness was both lower at middle elevations. However, Sheng et al. (2019) found that the forest soil fungal diversity decreased with the increase of elevation significantly. Similarly, Chen et al. (2022a) found that the richness of soil fungi decreased with the increase of elevation significantly. It was because the influence of elevation on the diversity of soil fungal communities was less than other factors (such as soil physical and chemical properties, etc.). Therefore, the diversity of fungi was related to the various and unpredictable influencing factors and it was necessary to increase species and environmental factors scope to deepen the understanding of biodiversity.

Alpha diversity patterns were found no regular trend along elevation (Hendershot et al., 2017). A possible explanation for this might be that the influence of local environmental variations on the soil fungal Alpha diversity were different along elevation (Zhao et al., 2022). Unfortunately, these findings were rather difficult to interpret because of the huge number of variable and unpredictable environmental factors. However the reason of it can be interpreted by statistical analysis with a huge number of research data (Zhang et al., 2022). Therefore, it is very important to carry out high‐density soil fungal diversity research in different elevations, different climates, and different soil characteristics.

One interesting finding was that we found the difference of the soil fungal Beta diversity at two local middle elevations (1400 and 1800 m) was small. On the contrary, Jamil et al. (2020) found the difference among samples at the medium elevation was large. This result was necessarily anticipated The different conclusions further confirmed the necessity of studying soil fungal diversity in different regions.

### Responses of soil fungi to environmental factors

Recent studies showed that various environmental factors affected the soil fungi. Todesco and Cronk (2017) found that the high vegetation diversity decreased the fungal species diversity because of a lack of special selective pressure. In the maritime Antarctic, the C/N ratio of soil was found one of the predictors of fungal Chao1 index (Newsham et al., 2016). Deng et al. (2020) found that the soil pH negatively influenced Chao1 index and ACE index.

Generally, the environmental factors and host distributions influenced the soil fungal community composition along elevation (Bayranvand et al., 2021; Jarvis et al., 2015). Environmental factors play a more important role than host distributions (Adamo et al., 2021). This study provided clear evidence that elevation, soil physical and chemical properties, and vegetation diversity jointly affected soil fungal community composition in the HLMNNR. As well as soil physical and chemical properties contributed the most, followed by elevation and vegetation diversity in the subtropical mountain forest. Yang et al. (2020) found that increasing soil pH decreased the soil microbial diversity. Effects of pH on fungal diversity and richness in alpine forests were significant (Liu et al., 2018). More generally, not the same, pH had some effects on soil fungal diversity and richness, but not significant, while pH had some significant effects on many fungi with high abundance in this study. In addition, between vegetation diversity and soil fungal richness or diversity, no significant correlation was found in this study. Steinauer et al. (2016) found vegetation diversity had positive effect on soil fungal richness. Heavy metals were also found a great influence on soil fungal diversity and richness, and significant correlations between soil metal ions and different fungal communities (Lin et al., 2020). This conclusion was same as the study in HLMNNR. We found that edaphic physical and chemical properties dominantly determine fungal diversity and composition. This was the same as the conclusion of Pan et al. (2023).

Global climate changes are bringing sharper extreme environmental conditions, with unknown consequences for soil microbial communities (Norby et al., 2016). In addition, all soils in earth are influenced by human disturbance, either directly by land use and land management or indirectly by the responses to the global changes of human‐induced such as pollution (Smith, 2005). In conclusion, more and more global change factors lead to directional changes in soil microbial communities (Rillig et al., 2019). There is an urgent demand to better understand the influences of different environmental factors on soil fungi in order to formulate microbial strategies to combat the extreme environmental conditions, such like climate warming and pollution (Jansson & Hofmockel, 2020). Here, our study increased the understanding of responses of soil fungi and soil entomopathogenic fungi to different environmental factors in HLMNNR, subtropical mountain forest. As environmental changes continue to intensify, it may be significant, especially in forest management.

### The variation in soil entomopathogenic fungi and responses of soil fungi to environmental factors

The relative abundance of *Isaria*, *Beauveria*, and *Metarhizium* was from large to small in turn was *Isaria*, *Metarhizium*, and *Beauveria* among the three entomopathogenic fungi concerned in this study. The relative abundance of *Beauveria* was particularly low, which was contrary to the results of Majchrowska‐safaryan and Tkaczuk (2021). The abundance of *Beauveria* was high in Slovakia, too (Medo & Ludovít, 2011). Inglis et al. (2019) found that the richness of *Metarhizium* decreased at higher elevation, while the richness of *Metarhizium* increased at high elevation in this study. The distribution of entomopathogenic fungi in forest soil is closely related to insect species. In nature, the species and richness of entomopathogenic fungi were closely related to the species and number of local insects (Dara et al., 2019). In this study, the diversity of *Metarhizium* was comparatively high, possibly because *Metarhizium* was more adaptable to slightly acidic soil than *Beauveria* (Issaly et al., 2005). In HLMNNR, slightly acidic soil (Table S1) was conducive to the presence of *Metarhizium*. As the limitations of the high‐throughput sequencing (Di Bella et al., 2013; Soergel et al., 2012) and the isolation technique of fungi (Posadas et al., 2012), the isolation result of soil fungi might not correspond to the result of high‐throughput sequencing completely (Deaver et al., 2019). Fortunately, at the genus level, the isolated entomopathogenic fungi could correspond to the entomopathogenic fungi found by high‐throughput sequencing in this study.

As mentioned earlier, Soil properties was found a key role in the distribution of entomopathogenic fungi (Qayyum et al., 2021). For example, organic soil had positive effect on the diversity of entomopathogenic fungi (Almeida et al., 2022). In this study, soil physical and chemical properties was found a greater contribution to both the richness and diversity of entomopathogenic fungi. This accords with previous observations. Specially, soil chemical properties like TP, AZn, and AFe played a key role in the distribution of entomopathogenic fungi.

Native strains increased the effectiveness of pest control (Uzman et al., 2019). While non‐native entomopathogenic fungal strains will decrease the effect and even bring some ecological risks (Goble et al., 2011). Therefore, the entomopathogenic fungi isolated from their natural environment were of great significance to biological control (Masoudi et al., 2018). We isolated many toxic entomopathogenic fungi in this study, in order to use them in biological control in the future. These entomopathogenic fungi we isolated were ‘native strains’, so they had high utilization value in HLMNNR. At the same time, it also proved that there were active entomopathogenic fungi in forest soil, and with suitable environmental conditions, insect epidemics caused by entomopathogenic fungi might break out.

The diversity and distribution of soil fungi was focused on by many studies (Chen et al., 2022b). Fully grasping the influencing factors can help to deeper understand the ecological function of soil fungi. Then various soil fungi will be better applied.

## CONCLUSION

The vertical distribution of forest soil fungi, entomopathogenic fungi, and their influencing factors in a subtropical mountain in western China were investigated and the relationship between environmental factors and soil fungal diversity was discovered in this study. *Isaria* was predominant at the equivalent taxonomic level of organization in the HLMNNR and was particularly presented at middle elevations. A greater richness of soil fungi was found at middle elevations, while a higher evenness of soil fungi was found at low elevation. Among all environmental factors, soil pH, AFe, AK, TK, and AZn were the most important influencing factors affecting the vertical distribution of fungi. Elevation, herb species diversity, and WS diversity were less important than soil physical and chemical properties. Many highly abundant soil fungi were also influenced by environmental factors. For soil entomopathogenic fungi, *Isaria* was predominant significantly followed by *Metarhizium* and *Beauveria*. *Isaria* showed a significant positive correlation with TP and AFe, while, AZn was negatively correlated significantly while E, pH, AP, and ACu showed a negative correlation with *Metarhizium*. However, SOC, TN, TK, AK, and AZn were negatively correlated with *Beauveria*. In general, the distribution and diversity of soil fungi and entomopathogenic fungi were greatly influenced by the physical and chemical properties of soil in HLMNNR. Totally 20 entomopathogenic fungi were isolated and were applied to insect pest control test. The virulence of isolated entomopathogenic fungi against larvae of *Tenebrio molitor* showed a mortality range from 31.11% to 100%. The above findings provide valuable data to deepen our understanding of the diversity of soil fungi, particularly the correlation between entomopathogenic fungi and environmental influencing factors in subtropical mountain forests.

## AUTHOR CONTRIBUTIONS

**Jiyang Zheng:** Data curation (equal); formal analysis (equal); investigation (equal); software (equal); validation (equal); visualization (equal); writing – original draft (lead); writing – review and editing (equal). **Jinduo Shi:** Data curation (equal); formal analysis (equal); investigation (equal); project administration (lead); resources (equal); validation (equal). **Dun Wang:** Conceptualization (lead); funding acquisition (lead); methodology (equal); project administration (lead); resources (lead); supervision (lead); validation (lead); writing – review and editing (lead).

## CONFLICT OF INTEREST STATEMENT

The authors declare no conflict of interest.

## Supporting information


Data S1

## Data Availability

All data generated or analysed during this study are included in this published article and its supporting information files. We have deposited the high‐throughput sequencing data in NCBI with accession number PRJNA701184.
