# Supplementary material for: Diversity of soil fungi and entomopathogenic fungi in subtropical mountain forest in southwest China
Source: Environ Microbiol Rep. 2024 Jun 29;16(4):e13267. doi: 10.1111/1758-2229.13267 (PMC11213981; doi:10.1111/1758-2229.13267)
Supplement: Supplementary file 1 — Data S1 [file EMI4-16-e13267-s001.doc]

**Table S1.** The environmental factors

|  | E | HS | WS | pH | SMC | SOC | TN | TP | TK | AP | AK | ACu | AZn | AFe | AMn | ECa | EMg |
| --- | --- | --- | --- | --- | --- | --- | --- | --- | --- | --- | --- | --- | --- | --- | --- | --- | --- |
| HLS22001 | 2197.80 | 16.00 | 12.00 | 5.81 | 54.78 | 38.88 | 2.72 | 0.99 | 20.89 | 4.90 | 169.00 | 1.09 | 5.67 | 32.32 | 144.49 | 12.22 | 1.30 |
| HLS22002 | 2234.00 | 8.00 | 5.00 | 5.04 | 62.43 | 45.57 | 3.22 | 0.99 | 17.04 | 7.13 | 177.00 | 1.10 | 7.65 | 34.26 | 155.88 | 8.79 | 1.15 |
| HLS22003 | 2202.20 | 17.00 | 12.00 | 5.18 | 66.78 | 41.35 | 3.13 | 1.33 | 16.17 | 6.53 | 171.00 | 0.92 | 7.87 | 28.47 | 140.74 | 11.57 | 1.27 |
| HLS22004 | 2206.00 | 13.00 | 3.00 | 5.01 | 56.08 | 43.27 | 3.13 | 1.06 | 15.04 | 5.23 | 181.00 | 0.90 | 6.28 | 33.76 | 137.05 | 9.38 | 1.05 |
| HLS22005 | 2180.20 | 8.00 | 5.00 | 4.89 | 67.35 | 48.18 | 3.28 | 1.05 | 14.01 | 5.33 | 154.00 | 1.04 | 7.48 | 34.71 | 146.35 | 8.66 | 1.09 |
| HLS18001 | 1852.40 | 16.00 | 5.00 | 7.76 | 39.11 | 21.95 | 1.50 | 1.33 | 21.33 | 6.47 | 83.00 | 1.01 | 1.51 | 42.92 | 6.22 | 6.70 | 1.70 |
| HLS18002 | 1830.20 | 14.00 | 9.00 | 7.99 | 32.93 | 19.19 | 1.15 | 0.91 | 25.65 | 5.17 | 74.00 | 1.21 | 2.57 | 35.42 | 9.89 | 5.51 | 0.73 |
| HLS18003 | 1822.00 | 20.00 | 9.00 | 7.84 | 22.61 | 15.20 | 1.01 | 1.20 | 22.99 | 8.07 | 69.00 | 1.01 | 2.33 | 38.79 | 10.96 | 8.83 | 1.15 |
| HLS18004 | 1809.60 | 16.00 | 5.00 | 7.72 | 25.51 | 16.90 | 1.49 | 0.75 | 23.49 | 8.60 | 91.00 | 1.25 | 2.92 | 36.61 | 8.40 | 9.08 | 1.85 |
| HLS18005 | 1804.20 | 13.00 | 4.00 | 7.28 | 41.12 | 19.77 | 1.20 | 1.22 | 28.11 | 6.03 | 72.00 | 0.81 | 2.14 | 42.34 | 12.81 | 9.36 | 1.77 |
| HLS14001 | 1344.60 | 16.00 | 5.00 | 6.95 | 75.14 | 53.44 | 4.10 | 3.09 | 25.82 | 13.07 | 160.00 | 1.02 | 4.26 | 45.14 | 179.83 | 30.76 | 3.51 |
| HLS14002 | 1487.60 | 27.00 | 16.00 | 7.15 | 70.64 | 54.36 | 4.31 | 3.31 | 28.18 | 12.47 | 163.00 | 1.42 | 5.63 | 55.38 | 155.84 | 20.34 | 2.52 |
| HLS14003 | 1507.60 | 15.00 | 5.00 | 6.48 | 67.35 | 45.05 | 3.80 | 2.04 | 30.97 | 17.80 | 143.00 | 0.96 | 5.21 | 47.59 | 186.12 | 23.63 | 1.91 |
| HLS14004 | 1492.60 | 21.00 | 12.00 | 6.83 | 83.14 | 60.00 | 4.60 | 2.77 | 21.59 | 12.73 | 144.00 | 0.10 | 4.89 | 46.50 | 192.99 | 33.92 | 2.73 |
| HLS14005 | 1537.00 | 16.00 | 6.00 | 7.65 | 70.59 | 46.56 | 4.15 | 2.61 | 29.59 | 10.80 | 156.00 | 0.84 | 3.16 | 38.23 | 152.38 | 24.50 | 2.17 |
| HLS10001 | 1032.40 | 15.00 | 6.00 | 7.83 | 48.73 | 47.15 | 4.45 | 0.78 | 15.59 | 22.27 | 162.00 | 2.66 | 14.54 | 29.81 | 167.80 | 23.41 | 2.32 |
| HLS10002 | 1010.00 | 16.00 | 7.00 | 6.56 | 54.72 | 68.01 | 4.87 | 0.68 | 15.31 | 28.60 | 151.00 | 3.96 | 17.02 | 26.38 | 177.62 | 21.99 | 2.08 |
| HLS10003 | 1035.60 | 13.00 | 5.00 | 6.17 | 60.97 | 53.69 | 5.95 | 0.97 | 11.15 | 17.13 | 159.00 | 3.40 | 17.32 | 24.14 | 151.05 | 33.17 | 2.30 |
| HLS10004 | 1020.20 | 14.00 | 5.00 | 5.90 | 56.87 | 59.58 | 3.95 | 1.19 | 11.07 | 21.77 | 143.00 | 2.60 | 18.99 | 28.07 | 166.34 | 31.56 | 1.62 |
| HLS10005 | 1029.00 | 18.00 | 11.00 | 6.24 | 61.31 | 58.42 | 6.37 | 0.57 | 11.27 | 25.87 | 156.00 | 2.80 | 21.57 | 31.23 | 163.61 | 29.13 | 2.20 |

*
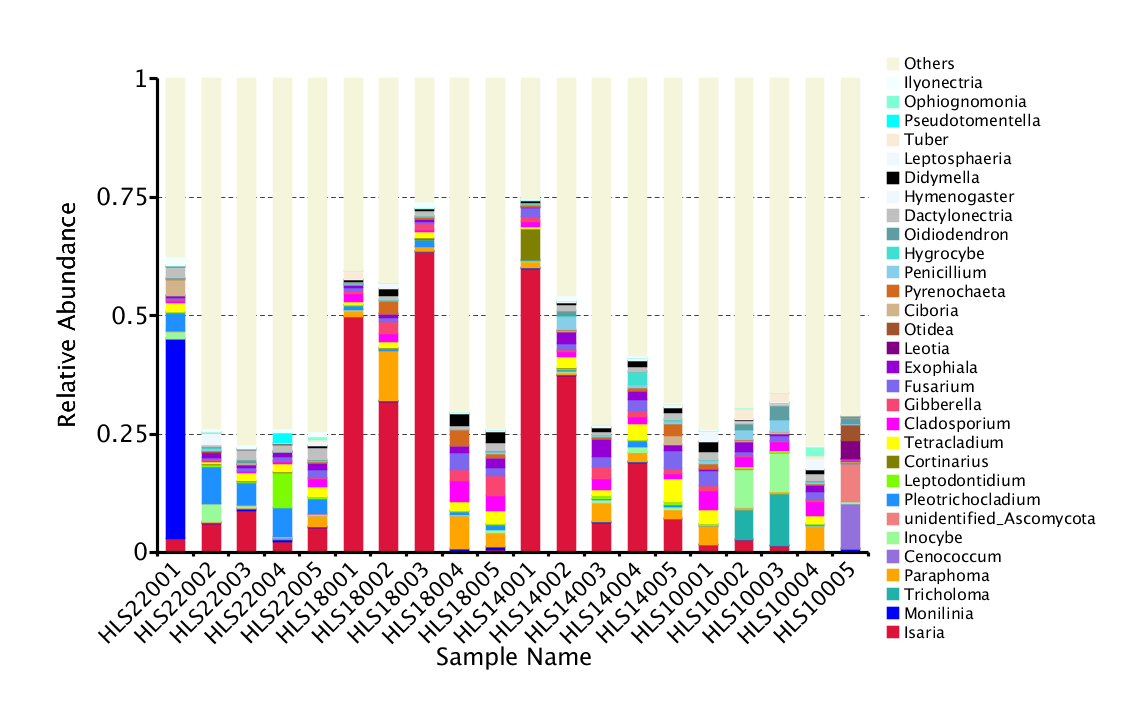
*

**Figure S1.** The Top30 genera fungal relative abundance histogram of ITS2 region for high-throughput sequencing of fungi with different soil sample in HLMNNR. HLS1000, elevation of 1000 m in HLMNNR; HLS1400, elevation of 1400 m in HLMNNR; HLS1800, elevation of 1800 m in HLMNNR; HLS2200, elevation of 2200 m in HLMNNR.


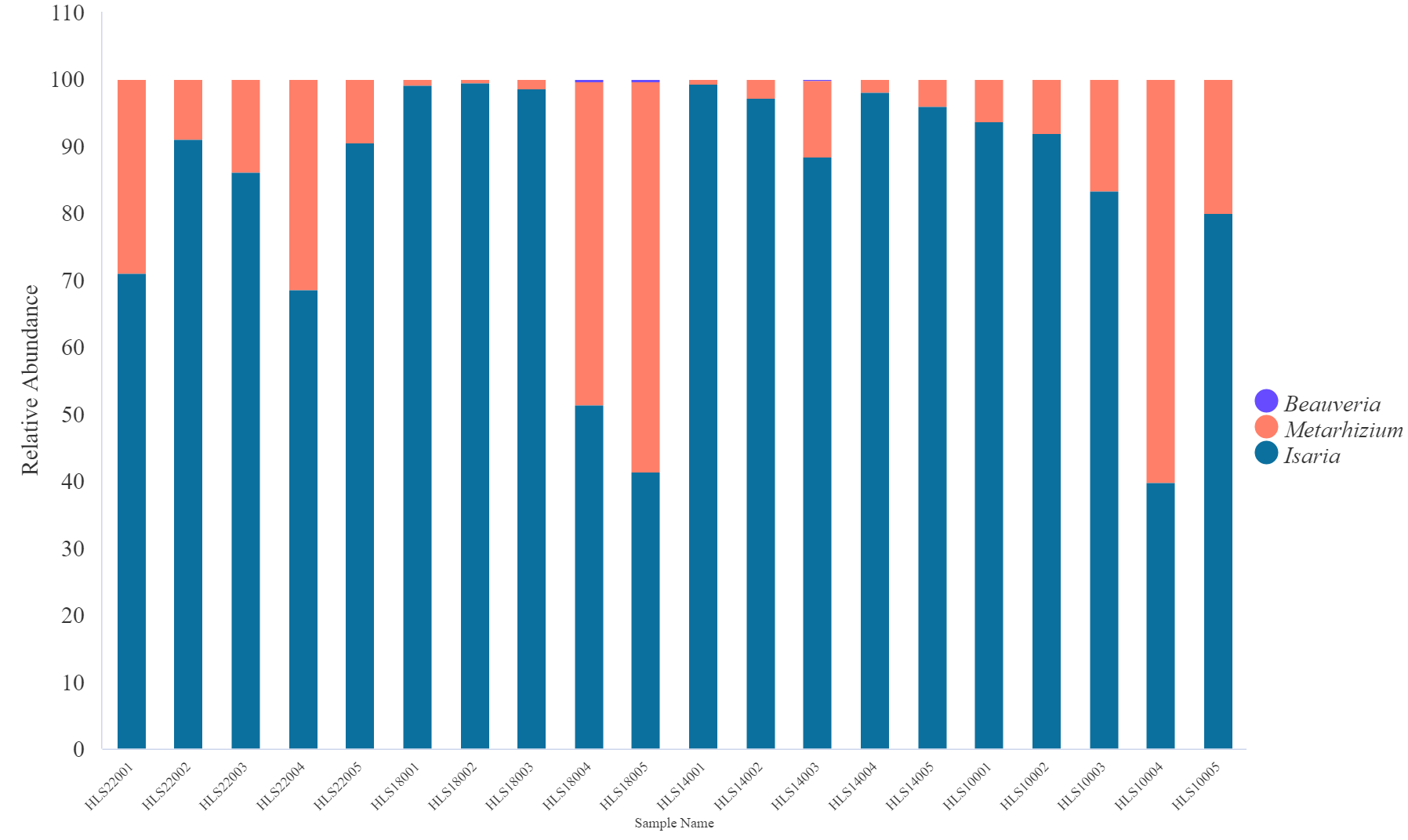


**Figure S2.** The Top30 genera entomopathogenic fungal relative abundance histogram of ITS2 region for high-throughput sequencing of fungi with different soil sample in HLMNNR. HLS1000, elevation of 1000 m in HLMNNR; HLS1400, elevation of 1400 m in HLMNNR; HLS1800, elevation of 1800 m in HLMNNR; HLS2200, elevation of 2200 m in HLMNNR.
